# Gut microbiota mediates positive effects of liraglutide on dyslipidemia in mice fed a high-fat diet

**DOI:** 10.3389/fnut.2022.1048693

**Published:** 2022-12-29

**Authors:** Li Zhao, Yue Qiu, Panpan Zhang, Xunan Wu, Zhicong Zhao, Xia Deng, Ling Yang, Dong Wang, Guoyue Yuan

**Affiliations:** ^1^Department of Endocrinology and Metabolism, Affiliated Hospital of Jiangsu University, Zhenjiang, Jiangsu, China; ^2^Department of Endocrinology and Metabolism, The First People’s Hospital of Lianyungang, Lianyungang, Jiangsu, China; ^3^Department of Endocrinology, Taicang Hospital of Traditional Chinese Medicine, Taicang, Jiangsu, China

**Keywords:** *Akkermansia*, dyslipidemia, glucagon-like peptide-1 receptor agonist, liraglutide, gut microbiota

## Abstract

Except for improving glycemic control, liraglutide, one of the glucagon-like peptide-1 receptor agonists, has exerted promising therapeutic effects for dyslipidemia. It has been proved that gut microbiota plays a dramatic role in regulating lipid metabolism. This study aims to explore whether liraglutide could improve dyslipidemia by modulating the gut microbiota in mice fed a high-fat diet (HFD). The C57BL/6 mice were fed a HFD to establish an animal model of dyslipidemia, and then administered with liraglutide or normal saline (NS) for 12 weeks. Indices of glucolipid metabolism were evaluated. Gut microbiota of the mice was analyzed by 16S rRNA gene sequencing. Compared with HFD group, liraglutide significantly alleviated weight, total cholesterol (TC) and low-density lipoprotein cholesterol (LDL) levels, meanwhile elevating high-density lipoprotein cholesterol (HDL) levels (all *p* < 0.05). The gut microbiota analysis revealed that liraglutide greatly reduced the relative abundance of *Firmicutes* and augmented that of *Bacteroidetes*, with a concomitant drop in the *Firmicutes*/*Bacteroidetes* ratio. Meanwhile, liraglutide dramatically changed the overall composition, promoted the growth of beneficial microbes (*Akkermansia*, *Lactobacillus*, *Parabacteroides*, *Oscillospira*, etc.), and inhibited the growth of harmful microbes (*AF12*, *Shigella, Proteobacteria*, *Xenorhabdus*, etc.). Especially, the relative abundance of *Akkermansia* increased the most after liraglutide treatment. Correlation analysis suggested that TC and LDL were positively correlated with some harmful bacteria, and negatively associated with beneficial bacteria. This study confirmed that liraglutide had a certain therapeutic effect on dyslipidemia in HFD-fed mice and could regulate the composition of the gut microbiota associated with lipid metabolism, especially *Akkermansia*. Thus, affecting gut microbiota might be a potential mechanism of liraglutide in attenuating dyslipidemia.

## Introduction

Dyslipidemia, including the elevation of serum total cholesterol (TC), total triglycerides (TG), and low-density lipoprotein cholesterol (LDL) as well as relative reduction of high-density lipoprotein cholesterol (HDL), is one of the worldwide prevalent health hazards. In China, the prevalence of dyslipidemia has exceeded 43%, according to 2019 statistics (1). Increasing evidences have emphasized that lipid metabolic disturbance, especially the imbalance of serum LDL and HDL levels, acts as a major risk factor for atherosclerotic cardiovascular disease (ASCVD), including hypertension, myocardial infarction, stroke, and sudden cardiac death (2–4). Thus, early detection and treatment of dyslipidemia are imperative to reduce cardiovascular events.

The widely used lipid-lowering drugs such as statins, fibrates, bile acid sequestrates and niacin, have shown great potential in preventing and treating dyslipidemia (5). Many synthetic anti-obesity medications have also been used to improve dyslipidemia, as obesity is an important risk for dyslipidemia; however, they all exert undesirable adverse reactions such as myopathy, pancreatitis (6), and transaminase elevations, as well as treatment resistance, which may hinder medication compliance (7, 8). Therefore, any treatment that safely lowers lipid levels is in great demand for the management of dyslipidemia and obesity.

Glucagon-like peptide-1 (GLP-1) is a gastric-derived anorexigenic peptide produced by intestinal enteroendocrine L cells mainly upon fat and carbohydrate intake. Once secreted, GLP-1 will be rapidly degraded by dipeptidyl peptidase-4 (DPP-4), therefore its half-life is only a few minutes (9). Liraglutide, one of the glucagon-like peptide-1 receptor agonists (GLP-1RAs), shares 97% of the amino acid sequence with endogenous human GLP-1, but has a prolonged half-life of 13 h because of its resistance to DPP-4 degradation. Although liraglutide mainly improves blood glucose levels, recent data has also revealed it has a beneficial effect on cardiovascular outcomes (10, 11), by modulating other risk factors, such as dyslipidemia (12), blood pressure (13), and endothelial dysfunction (14). Liraglutide is also the first GLP-1RA approved for treating obesity in both type 2 diabetes mellitus (T2DM) and non-T2DM patients (15), because it has the advantage that its weight loss and lipid-lowering efficacy is independent of hypoglycemic mechanisms.

Additionally, gut microbiota has been reported to play a crucial role in the maintenance and establishment of human health (16). A lager number of studies have shown that altered gut microbiota composition has been associated with dyslipidemia (3, 17) and cardiometabolic diseases (18). Increasing the abundance of some beneficial bacteria could improve dyslipidemia. Therefore, modulating gut microbiota may be a beneficial strategy to prevent the development of dyslipidemia (19). On the other hand, there is evidence that GLP-1 may change the composition of gut microbiota by influencing the biological function of intestinal epithelium (20). Further, GLP-1RAs are known to affect the intestinal environment and change the gut microbiota (21, 22). For example, liraglutide was reported to control glucose-induced insulin secretion partly through influencing the gut microbiota and the intestinal immune system (23). Its beneficial effect on weight loss *via* modulating the structure of gut microbiota was also confirmed in both diabetic obese and simple obese rodents (22, 24). Therefore, we speculated that liraglutide might also be able to improve dyslipidemia by modulating the gut microbiota.

To this aim, we treated high-fat diet (HFD)-fed mice with liraglutide or vehicle (saline solution) for 12 weeks. At the end of the experiment, 16S rRNA gene sequencing was used to analyze the gut microbiota of the mice. The change of gut microbiota contributed to the beneficial effects of liraglutide against dyslipidemia, which might serve as a potential therapeutic target for dyslipidemia.

## Materials and methods

### Animals and the experimental design

A total of 24 male C57BL/6 mice (4 weeks old), provided by the Laboratory Animal Research Center of Jiangsu University (Zhenjiang, China), were randomly assigned to two groups: normal control group (NC, *n* = 8) and HFD group (*n* = 16). These mice were singly housed in a specific-pathogen-free (22 ± 2°C) environment with a 12/12-h light/dark cycle and could free access to a standard laboratory diet and water. After 1 week of adaptation, the NC group was administered a standard diet, whereas the HFD group was administered a HFD (37% carbohydrate, 18% protein, and 45% fat) for 12 weeks to induce dyslipidemia. Then, the HFD group was further randomly subdivided into the HFD group (*n* = 8) and the HL group (HFD + liraglutide, *n* = 8). The HL group received a daily subcutaneous injected with liraglutide (Victoza, Novo Nordisk, Denmark, 0.2 mg/kg body weight), while the NC and HFD groups were injected with an equal volume of NS for another 12 weeks. Fasting body weights were monitored every 2 weeks. After overnight fasting, all the mice were sacrificed at the 24th week, and the blood and fecal samples were collected, which were stored at−80°C until the next analysis. The day before sacrifice, all mice were fasted overnight and the intraperitoneal insulin tolerance test (IPITT) was carried out.

### Biochemical analysis

Fasting blood glucose (FBG) was measured by chemiluminescence method, and automatic biochemical analyzer was used to detect lipid profile. All animal experiments were performed according to protocols (UJS-IACUC-2020032535) approved by the Animal Research Committee at the Institute of Laboratory Animals, Jiangsu University Medical College.

### Fecal DNA extraction and 16S rRNA gene sequencing

Microbial DNA of fecal samples was extracted by using the Quant-iT PicoGreen dsDNA Assay Kit (Invitrogen, Carlsbad, CA, USA), which was then used as the template to amplify the V3 and V4 hypervariable regions of ribosomal 16S rRNA genes by PCR (25 cycles, Initial denaturation 98°C for 2 min→Denaturation 98°C for 15 s→Annealing 55°C for 30 s →Extension 72°C for 30 s→Final extension 72°C for 5 min). The specific forward primer was 338F 5′-ACTCCTACGGGAGGCAGCA-3′ and the reverse primer was 806R 5′-GGACTACHVGGGTWTCTAAT-3′. All PCR were performed in triplicate with 25 μl reaction mixture containing 5 μl of 5× reaction Buffer, 5 μl of 5× GC Buffer, 2 μl of 2.5 mM dNTPs, 1 μl of each primer (10 μM), 0.25 μl of Q5 DNA Polymerase, and 2 μl of template DNA. The amplified PCR products were extracted on 1.20% agarose gels and purified. 16S rRNA gene sequencing was performed using the Illumina NovaSeq-PE250 platform at Shanghai Personal Biotechnology Co., Ltd. (Shanghai, China) to obtain 2 × 480 bp paired-end reads.

### Statistical analysis

Data analyses were conducted with SPSS version 26.0 (SPSS Inc., Chicago, IL, USA). Data were presented as means ± standard error of the mean (SEM), and statistical significance among the three groups was analyzed by one-way analysis of variance (ANOVA). The raw data obtained by sequencing were stored in FASTQ format and performed with QIIME 2 (2019.4) (25). Sequences were then quality filtered, denoised, merged, and chimera removed using the DADA2 plugin (26). Amplicon sequence variants (ASVs) were aligned with mafft (27) and used to construct a phylogeny with fasttree2 (28). Alpha-diversity metrics (Chao1, Observed species, Shannon, and Simpson), and beta diversity metrics [weighted UniFrac (29), unweighted UniFrac (30), Jaccard distance, and Bray–Curtis dissimilarity] were estimated using the diversity plugin to reflect community richness and diversity. Principal coordinates analysis (PCoA) and principal components analysis (PCA) were then performed based on the matrix of beta diversity distance to study the differences between different microbial communities. Relative abundance of bacteria was assessed by the linear discriminant analysis effect size (LEfSe). Spearman’s correlation analysis was performed to explore relationship between metabolic parameters and gut microbiota composition. The area under the curve (AUC) of blood glucose in IPITT was calculated. Statistical difference was set as *p* < 0.05 value.

## Results

### Liraglutide suppressed body weight gain and ameliorated lipid profile

**FIGURE 1:**
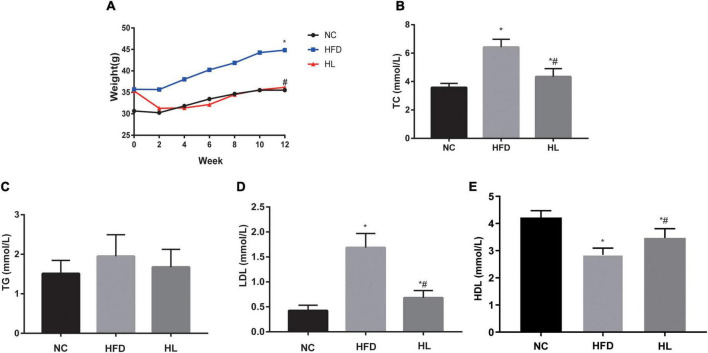
Liraglutide attenuated weight gain and blood lipid profile. **(A)** Body weight. **(B)** Total cholesterol (TC) levels. **(C)** Triglyceride (TG) levels. **(D)** Low-density lipoprotein (LDL) cholesterol levels. **(E)** High-density lipoprotein (HDL) cholesterol levels. Data are presented as the mean ± SEM. *p < 0.05 vs. NC group; ^#^*p* < 0.05 vs. HFD group.

The body weights of mice in all the three groups showed an overall tendency to increase as the experiment went on. After the injection of liraglutide, significant weight loss was observed within the first 2 weeks. During the period of administration, the body weights of the HL group were basically close to those of NC group (*p* > 0.05), while the average body weight of the HFD group was the highest among the three groups (*p* < 0.05). At the end of experiment, the weights of the HL group were decreased obviously (Figure 1A, *p* < 0.05).

The key biochemical parameters of serum were monitored to explore the impact of liraglutide on dyslipidemia. Compared with the HFD group, supplementation with liraglutide dramatically attenuated the lipid profile levels, including TC, TG and LDL (Figures 1B–D). Especially, TC and LDL levels in HL group were statistically significant compared with the other two groups (Figures 1B,D, *p* < 0.05). Contrarily, HDL levels were decreased greatly in HFD group, but increased dramatically after liraglutide treatment (Figure 1E, *p* < 0.05).

### Liraglutide improved insulin sensitivity

**FIGURE 2:**
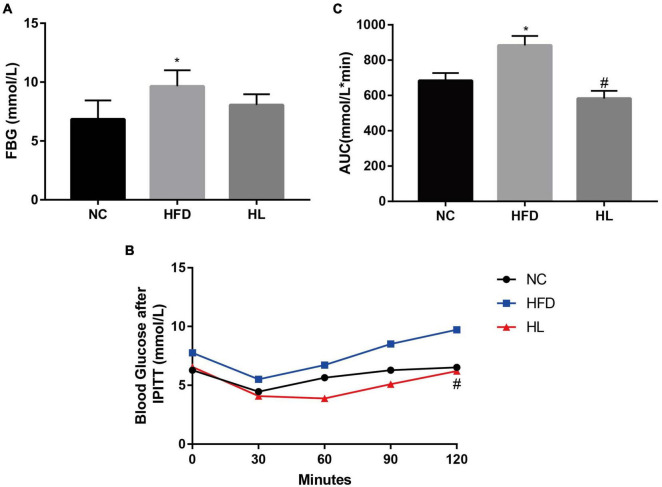
Liraglutide reduced FBG and improved insulin sensitivity in HFD-fed mice. **(A)** Fasting blood glucose (FBG) levels. **(B)** Blood glucose after intraperitoneal insulin tolerance test (IPITT). **(C)** The area under the curve (AUC) of IPITT. Data are presented as the mean ± SEM. *p < 0.05 vs. NC group; ^#^*p* < 0.05 vs. HFD group.

It could be clearly observed that the average FBG level of the HFD group was generally higher than those of the NC and HL groups, with statistically significant difference between the HFD group and the NC group (Figure 2A, *p* < 0.05). There was no difference of FBG levels between the HL group and the NC group (Figure 2A, *p* > 0.05), indicating that liraglutide could attenuate body weight gain and improve dyslipidemia without increasing the risk of hypoglycemia. The results of IPITT showed that compared with the HFD group, liraglutide apparently improved the 5-point blood glucose levels (Figure 2B, *p* < 0.05). Although the blood glucose level of the HL group at 0 min was slightly higher than that of the NC group, the blood glucose levels at 30, 60, 90, and 120 min after intraperitoneal insulin administration were lower than those in the NC group. Meanwhile, the AUC of blood glucose levels was the lowest in the HL group (Figure 2C, *p* < 0.05). These results demonstrated that liraglutide could greatly increase insulin sensitivity in HFD-fed mice.

### Liraglutide altered the structure of gut microbiota in high-fat diet-fed mice

**FIGURE 3:**
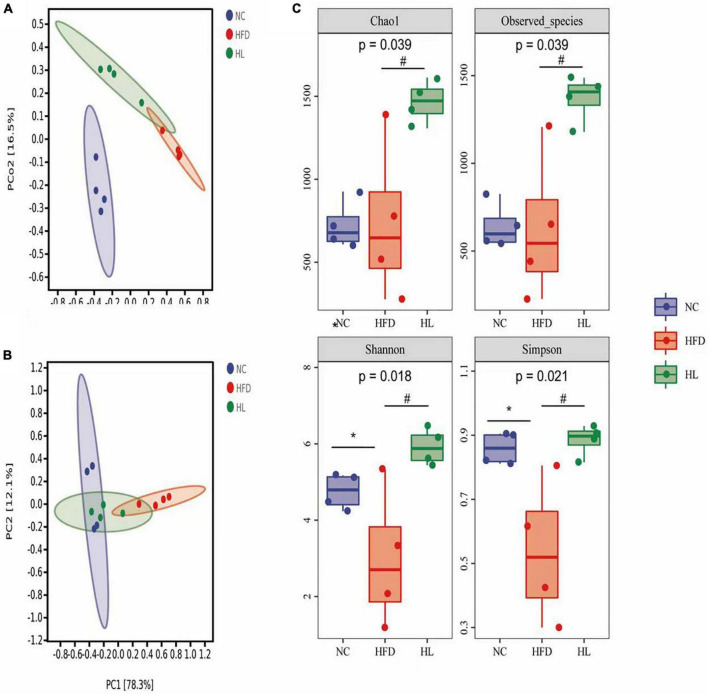
Liraglutide altered the overall structure of gut microbiota in HFD-fed mice. **(A)** Principal coordinates analysis (PCoA). **(B)** Principal components analysis (PCA). **(C)** Alpha-diversity metrics (Chao1, Observed_species, Shannon, and Simpson). Data are presented as the mean ± SEM. *p < 0.05 vs. NC group; ^#^*p* < 0.05 vs. HFD group.

The PCoA and the PCA plots levels suggested greatly different structural patterns between the three groups (Figures 3A,B). It was noteworthy that the dots of HL group were close to the NC group, revealing that after liraglutide invention, the diversity of the gut microbiota was similar with that of the NC group. This means that liraglutide treatment could partially reversed the changes in the gut microbiota caused by HFD in mice.

As illustrated in Figure 3C, Chao1 and Observed species in the HL group were significantly higher than those of the HFD group (*p* < 0.05). Compared with the NC group, Shannon and Simpson indices in the HFD group were also dropped, while the values of the HL group were significantly increased (*p* < 0.05). This indicated that liraglutide treatment could contribute to an increase in both diversity and richness of the gut microbiota.

### Liraglutide improved the microbial composition in high-fat diet-fed mice

**FIGURE 4:**
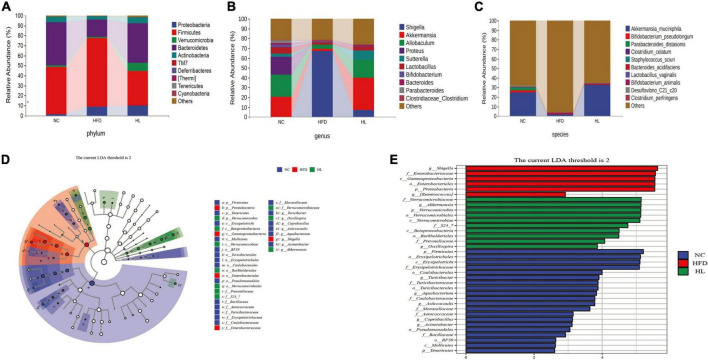
Liraglutide changed the composition of gut microbiota in HFD-fed mice. **(A)** Composition of gut microbiota at the phylum level. **(B)** Composition of gut microbiota at the genus level. **(C)** Composition of gut microbiota at the species level. **(D)** Linear discriminant analysis effect size (LEfSe) cladogram. **(E)** Linear discriminant analysis effect size (LEfSe) histogram.

At the phylum level (Figure 4A), high-fat feeding obviously augmented the relative abundance of *Firmicutes* and reduced the relative abundance of *Bacteroidetes*, thus the ratio of *Firmicutes* to *Bacteroidetes* (F/B) increased accordingly. Inversely, liraglutide administration significantly reversed the relative abundance of *Firmicutes* and *Bacteroidetes*, with a concomitant drop in the F/B ratio. At the genus level (Figure 4B), the abundance of *Akkermansia, Lactobacillus, Parabacteroides, Oscillospira, Sutterella*, and *Allobaculum* were suppressed by HFD, while their growth was promoted by liraglutide. Especially, *Akkermansia* was significantly enriched in the HL group. Further, at the species level, *Akkermansia muciniphila* had the most variable proportion of the whole species, which was 25.13% in the NC group, 1.90% in the HFD group, and 33.18% in the HL group. This result also showed that liraglutide greatly augmented the abundance of *A. muciniphila* (Figure 4C).

Additionally, the LEfSe method was applied to explore the biomarkers of dyslipidemia in gut microbiota (Figures 4D,E). In total, there were 20, 6, and 10 obviously different OTUs in NC, HFD, and HL group, respectively (Figure 4E). Cladogram analysis also supported the different levels of labeled taxa obtained from the LEfSe in the experimental groups (Figure 4D). After liraglutide treatment, the HL group was enriched in Verrucomicrobia at the phylum level, Verrucomicrobiae and Betaproteobacteria at the class level, Verrucomicrobiales and Burkholderiales at order level, Verrucomicrobiaceae and Prevotellaceae at the family level, and *Akkermansia* at the genus level. It is worth noting that *Akkermansia* belongs to the family Verrucomicrobiaceae, and further to the order Verrucomicrobiales, class Verrucomicrobiae, and phylum Verrucomicrobia.

### Relationship between gut microbiota composition and metabolic parameters

**FIGURE 5:**
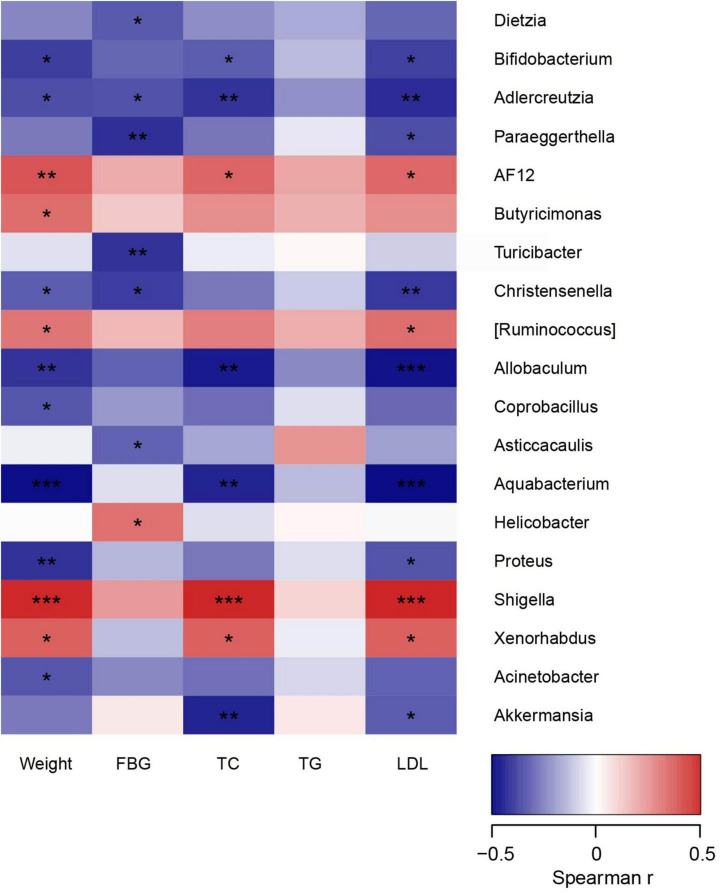
The relationship between microbiota composition and metabolic parameters. *p < 0.05, **p < 0.01, and ***p < 0.001.

In order to comprehensively analyze the correlation between metabolic parameters and the composition of intestinal microflora, Spearman’s correlation analysis was performed. As shown in Figure 5, *AF12*, *Shigella*, and *Xenorhabdus* were positively correlated with weight and lipid profile, while *Bifidobacterium*, *Adlercreutzia, Allobaculum*, and *Aquabacterium* were found to be negatively related to weight and lipid profile (*p* < 0.05). Besides, *Akkermansia* was negatively associated with TC and LDL (*p* < 0.05). Interestingly, these negatively related strains were enriched in the intestine of mice in HL group, but deficient in HFD group. Thus, it could be supposed that these related microbiota stains might be essential factors for the beneficial effect of liraglutide on dyslipidemia.

## Discussion

The pathophysiology of dyslipidemia is very complex, which has been only partially elucidated. In this study, liraglutide treatment could effectively attenuate body weight and lipid profile levels in mice with HFD-induced dyslipidemia. Meanwhile, liraglutide mainly exerted the hypolipidemic effect by modulating the composition of gut microbiota, especially by increasing the abundance of *Akkermansia*.

There are tens of thousands of microorganisms colonizing the human intestinal tract, which can help to break down the indigestible food. Previous studies have revealed that the gut microbiota accounts for 30% of the energy absorption of the host (31, 32). Besides, it carries out varieties of metabolic activities in the body of the host and produces a range of metabolites which further affect human’s health (33). The relationship between the gut microbiota and metabolic diseases, especially obesity, is one of the main foci of research in recent years. Emerging evidence has clarified that gut microbiota can regulate the lipid metabolism, which is closely associated with dyslipidemia (17, 19, 34).

In recent decades, the gut microbiota, dominated by *Bacteroidetes* and *Firmicutes*, has been reported to have a critical impact on the development of obesity (19, 34). *Firmicutes/Bacteroidetes* (F/B) ratio is also commonly used to reflect the changes in microbial structure at the phylum level and is used as a marker to measure overweight and obesity (35). The HFD can significantly decrease the relative abundance of *Bacteroides* and increase the abundance of *Firmicutes* (34), resulting in gut microbiota dysbiosis. The dysbiosis, in turn, further disrupts the normal lipid metabolism and then causes dyslipidemia, forming a vicious cycle. Many researches demonstrate that a higher F/B ratio has a positive correlation with obesity in obese mice and patients (34) (36). When obese subjects reduced their intake of HFD and lost weight, the proportion of *Bacteroidetes* increased miraculously (37). Similar results were also confirmed in ob/ob mice and HFD-induced obese (DIO) mice (38, 39). Our study revealed that the abundance of *Firmicutes* increased and *Bacteroides* decreased at the phylum level in HFD group, which was similar with the above results. After liraglutide intervention, the proportion of *Firmicutes* was dramatically reduced, while the relative abundance of *Bacteroides* was greatly augmented. The ratio of F/B was then accordingly decreased after liraglutide treatment.

To further evaluate precise changes in the gut microbiota, we analyzed the microbiota differences at other taxonomic levels. Compared with the NC group, HFD reduced the abundance of *Akkermansia, Lactobacillus, Parabacteroides, Oscillospira, Sutterella*, and *Allobaculum*, etc. After 12 weeks of liraglutide administration, the relative abundance of these genera was dramatically increased, especially the proportion of *Akkermansia* having the greatest change. Many studies have shown that the above strains are negatively related to obesity, and they are also referred to as thin bacteria (40, 41). For example, the well-known *Lactobacillus* genus is typically one of the top three probiotics, and its abundance is inversely related to the growth of body weight and fat mass (42). Kim et al. found that long-term oral administration of *Lactobacillus* gasseri BNR17 contributed to the reduction of visceral fat in obese adults (43); Jang et al. elucidated that oral administration of a variety of *Lactobacillus* inhibited HFD-induced NF-κB activation and increased the activation of AMP-activated protein kinase and SIRT-1 expression in liver, so as to reduce obesity and relieve inflammatory symptoms (44). *Oscillospira* is another beneficial bacterium, which is strongly correlated with leanness and obesity, as well as human health (34). The abundance of *Oscillospira* was discovered to be decreased significantly in patients with non-alcoholic steatohepatitis, which had a negative correlation with BMI and inflammatory indicators (45). In addition, numerous evidences demonstrate that *Oscillospira* plays a critical role in many metabolic activities that are associated with dyslipidemia and obesity (34) (46). *Paracobacteria* has far-ranging cholic acid conversion functions, hydrolyzing kinds of bound bile acids and converting them to secondary bile acids (such as ursodeoxycholic acid and lithocholic acid). These secondary bile acids play a role in improving lipid metabolism disorders *via* activating intestinal FXR signaling pathway (47).

As mentioned above, the proportion of *Akkermansia* in the HL group enriched the most. When analyzing the difference between species at different taxonomic levels, we found that *Verrucomicrobia, Verrucomicrobiaes, Verrucomicrobiaceae*, and *Akkermansia* in the feces of HL group mice had relatively high LDA values, suggesting that these taxa might be used as the marker species. In the further analysis of the level of microflora, the result showed that *A. muciniphila* had the largest change in the proportion of the whole species, which was nearly 18 times of HFD group. What is more, *A. muciniphila* belongs to the genus *Akkermansia*, which further belongs to family *Verrucomicrobiaceae*, order *Verrucomicrobiales*, class *Verrucomicrobiae*, and phylum *Verrucomicrobia*. Therefore, *A. muciniphila* might play a key role in the effects of weight loss and lipid-lowering mediated by liraglutide. *Akkermansia*, a strictly anaerobic Gram-negative enteric bacterium first isolated and proposed by Derrien et al. in 2004 from human feces, is non-motile and usually found in 1–4% abundance in the gut (48). *Akkermansia* has the specific function of degrading intestinal epithelial mucins and can use mucins as sole carbon and nitrogen sources for growth, with the main metabolites being oligosaccharides and short chain fatty acids (49). Although *Akkermansia* is associated with metabolic diseases and beneficial effects have been reported in host metabolism, its molecular mechanism has not been identified. Some researchers have found that *Akkermansia* can reduce the expression of genes involved in fatty acid synthesis and transport in both liver and muscle, alleviate endoplasmic reticulum stress, and thus improve lipid accumulation and metabolic disorders (50). *Akkermansia* was also found to increase thermogenesis and GLP-1 secretion in HFD- fed C57BL/6 mice by inducing uncoupling protein 1 in brown adipose tissue and GLP-1 secretion throughout the body (51). In this study, *Akkermansia* was reported to be negatively correlated with TC and LDL. Thus, we speculated that *Akkermansia* might be a key specie to promote the reduction of lipid levels, and liraglutide might reduce hyperlipidemia in HFD-fed mice by enriching the abundance of *Akkermansia*.

In the current study, liraglutide not only promoted the growth of beneficial bacteria, but also greatly inhibited the growth of harmful bacteria such as *AF12, Shigella*, *Proteobacteria*, etc. Spearman correlation analysis showed that these genera were also positively correlated with body weight and lipid profile. *AF12* is a poorly investigated taxon, which was reported to be enriched in obese mice (52). *Shigella* is a kind of Gram-negative pumilobacter, which is the typical intestinal pathogen of human bacterial dysentery. All strains of *Shigella* have strong endotoxin, like lipopolysaccharide (LPS), which can combine with toll-like receptor 4 to activate a variety of cell signaling pathways, induce chronic subclinical inflammation, and make the intestinal wall permeability increased, thus promoting the absorption of toxins. Then, those toxins act on the central nervous system and cardiovascular system, causing a series of clinical toxemia symptoms, such as fever, mental disturbance, and even toxic shock (53). Data from different sources indicate that the increase of circulating LPS level helps to explain why the relative abundance of *Shigella* is significantly increased in both obese rodents and humans (54, 55). Additionally, as a potential diagnostic biomarker of gut microbiota dysbiosis, the increased abundance of *Proteobacteria* was reported to be correlated with metabolic disorders (52, 56). The abundance of these harmful bacteria was significantly decreased by liraglutide, indicating its potential effect on preventing dyslipidemia and the related metabolic endotoxemia.

Another interesting finding in this study was that liraglutide increased the abundance of *Sutterella*, which was negatively associated with inflammation (57). Contrarily, the prevalence of *Proteobacteria* was markedly attenuated by liraglutide, as the increased relative abundance of *Proteobacteria* was found to be positively related to inflammation (56). Accumulating evidence suggests that inflammation is a critical and reversible mechanism by which obesity promotes the progression of the inflammatory diseases such as T2DM, NAFLD, dyslipidemia, and various types of cancer (58, 59). Inflammation induced by HFD is a contributing factor to metabolic disorders, and local hyperlipidemia is also speculated to be strongly associated with adipocyte death, altered adipose tissue function, and chronic low-grade inflammation (60). However, to clarify whether liraglutide breaks the link between inflammation and dyslipidemia through regulating related gut microbiota, we need to do further experiments.

The innovation of this study is that we put forward that the growth of *Akkermansia* might be closely related to liraglutide mediating weight loss and lipid reduction. However, whether transplantation of the gut microbiota of mice after liraglutide intervention into HFD-fed mice could also display similar lipid-lowing function needs to be further explored. Another limitation is that this study only conducted a single dosage of liraglutide and did not explore the suitable dose range and potential therapeutic relevance, which is incomplete. Thus, we need further studies to do so verify this phenomenon.

## Conclusion

In summary, liraglutide could prevent HFD-induced dyslipidemia, and mainly exerted the hypolipidemic effect by modifying the structure and composition of gut microbiota in HFD-fed mice. As the relative abundance of *Akkermansia* increased the most after liraglutide treatment, we speculated that *Akkermansia* might play a key role in liraglutide’s lipid-lowering effect. To confirm this hypothesis, further investigations are needed.

## Data availability statement

The original contributions presented in the study are publicly available. This data can be found here: https://figshare.com/s/ebdc21b93632da6a0387.

## Ethics statement

This animal study was reviewed and approved by the Biomedical Research Ethics Committee of Affiliated Hospital of Jiangsu University, Zhenjiang, China.

## Author contributions

LZ and GY conceived and designed the study. LZ and YQ performed the analysis, prepared the figures, and drafted the manuscript. YQ, PZ, ZZ, XD, and XW performed the experiment and collected the data. LY, DW, and GY conceived the study and participated in its design and coordination. All authors discussed the results and approved the final manuscript.

## Abbreviations

GLP-1RA: glucagon-like peptide-1 receptor agonists
HFD: high-fat diet
FBG: fasting blood glucose
TG: triglycerides
TC: total cholesterol
LDL: low-density lipoprotein
T2DM: type 2 diabetes mellitus
AUC: area under the curve
IPITT: intraperitoneal insulin tolerance test.
